# Acute Myocardial Infarction in a Young Male With COVID-19 Infection

**DOI:** 10.7759/cureus.35044

**Published:** 2023-02-16

**Authors:** Hovra Zahoor, Eboselumen Aigbe, Aamir Hussain, Hema Vankayala

**Affiliations:** 1 Internal Medicine, HCA Florida Orange Park Hospital, Orange Park, USA; 2 Hematology and Oncology, National Institute of Health, Baltimore, USA; 3 Hematology and Oncology, HCA Florida Orange Park Hospital, Orange Park, USA

**Keywords:** acute coronary thrombosis, venous thromboembolsim, mechanical thrombectomy (mt), covid-19, acute myocardial infarction

## Abstract

Coronavirus disease 2019 (COVID-19) infection is a global health crisis resulting in significant morbidity and mortality. The presentation of COVID-19 infection is variable, ranging from an asymptomatic carrier state to multi-organ failure. While cases of COVID-related myocarditis and myocardial dysfunction are well reported, only a few cases of coronary artery thrombosis resulting in myocardial infarction are noted on literature review. However, the previously reported cases were in patients with high risk for coronary artery disease. We hereby report a case of a young man with no significant past medical history or cardiovascular risk factors who presented with severe chest pain and was diagnosed with acute myocardial infarction in the setting of COVID-19 infection requiring intervention. We want to report this case to improve awareness in the community about COVID-related arterial thrombosis and have a high index of suspicion for this regardless of the person’s risk factors for cardiovascular diseases.

## Introduction

Coronavirus disease 2019 (COVID-19) infection is a global health crisis, described as a pandemic by the World Health Organization in March 2020 [1]. Clinical presentation of COVID-19 is variable and includes an asymptomatic form, upper respiratory tract symptoms and severe disease characterized by acute hypoxic respiratory failure requiring mechanical ventilation, septic shock and multi-organ dysfunction. Complications of COVID-19 include gastrointestinal, cardiovascular, thromboembolic and neurological sequelae. Thrombotic events occur in about 5-23% of cases [2]. Venous thromboembolism in patients with COVID-19 is well established and arterial thrombosis has also been reported. However, while cases of acute myocardial infarction in patients with COVID-19 infection have been reported, these patients exhibited either pre-existing coronary artery disease or had high risk factors for coronary artery disease. Here we present a case of a 27-year-old male with no past medical history of cardiovascular diseases and no significant risk factors for coronary artery disease who was found to have a right coronary artery thrombus resulting in acute myocardial infarction arising in the setting of COVID-19 infection.

## Case presentation

A 27-year-old male with stage 1 obesity and no significant past medical problems presented with sudden onset of severe, substernal chest pain which awakened him from sleep. He also experienced diaphoresis, nausea and vomiting with chest pain. He denied use of tobacco products or recreational drugs. No personal history of similar symptoms and no family member suffered from premature coronary artery disease.

**Figure 1:**
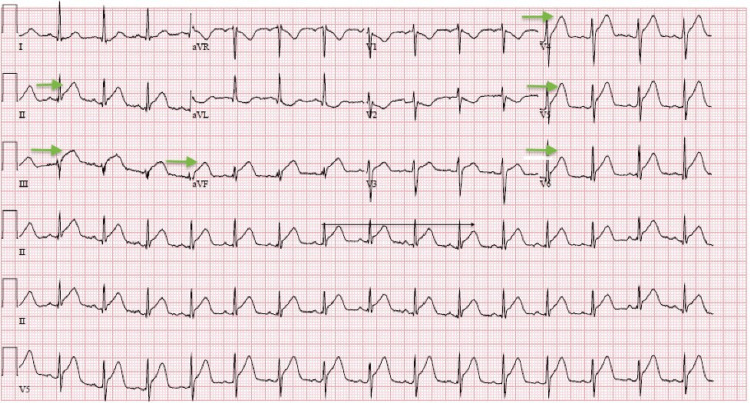
EKG showed ST elevation in infero-lateral leads (as depicted by arrows)

Patient was evaluated in the emergency department. On arrival, he was found to have a temperature of 98.7 F, blood pressure of 136/68 mmHg, heart rate of 92 beats per minute and respiratory rate of 18 breaths per minute. His pulse oxygen saturation (SpO2) was 97% on room air. Initial laboratory work-up was remarkable for positive COVID-19 nucleic acid amplification test. Complete blood count and complete metabolic panel were within normal range with the exception of hypokalemia; potassium of 2.9 mmol/L (normal range: 3.7-5.9 mmol/L). Lipid profile included a total cholesterol of 131 mg/dL (normal range <200 mg/dL), low-density lipoprotein (LDL) of 91.5 mg/dL (normal range 0-130 mg/dL), high-density lipoprotein (HDL) of less than 20 mg/dL (normal range 40-59 mg/dL) and a triglyceride level of 210 mg/dL (normal range <150 mg/dL). Urine drug screen was positive for cannabinoids and the patient denied any other illicit drug use. Troponins were negative. Electrocardiogram (EKG) on arrival showed ST elevation in the inferior and lateral leads (Figure 1), which prompted emergent cardiology consultation.

The patient was emergently taken to the catheterization laboratory. Cardiac catheterization revealed 100% thrombotic occlusion of the right coronary artery requiring pharmaco-mechanical thrombectomy and placement of two drug-eluting stents. It is to be noted that the remainder of the coronary vasculature exhibited minimal coronary artery disease. Patient was also started on aspirin 81 mg daily, prasugrel 10 mg daily, and atorvastatin 80 mg daily; he also did receive an intravenous tirofiban infusion at 18.4 ml/hr for 16 hours.

Once stabilized, patient admitted his COVID-19 unvaccinated status and that he did suffer from cough, fevers and shortness of breath for two weeks that dissipated several days before presentation. Patient during this hospitalization exhibited no respiratory symptoms.

**Figure 2:**
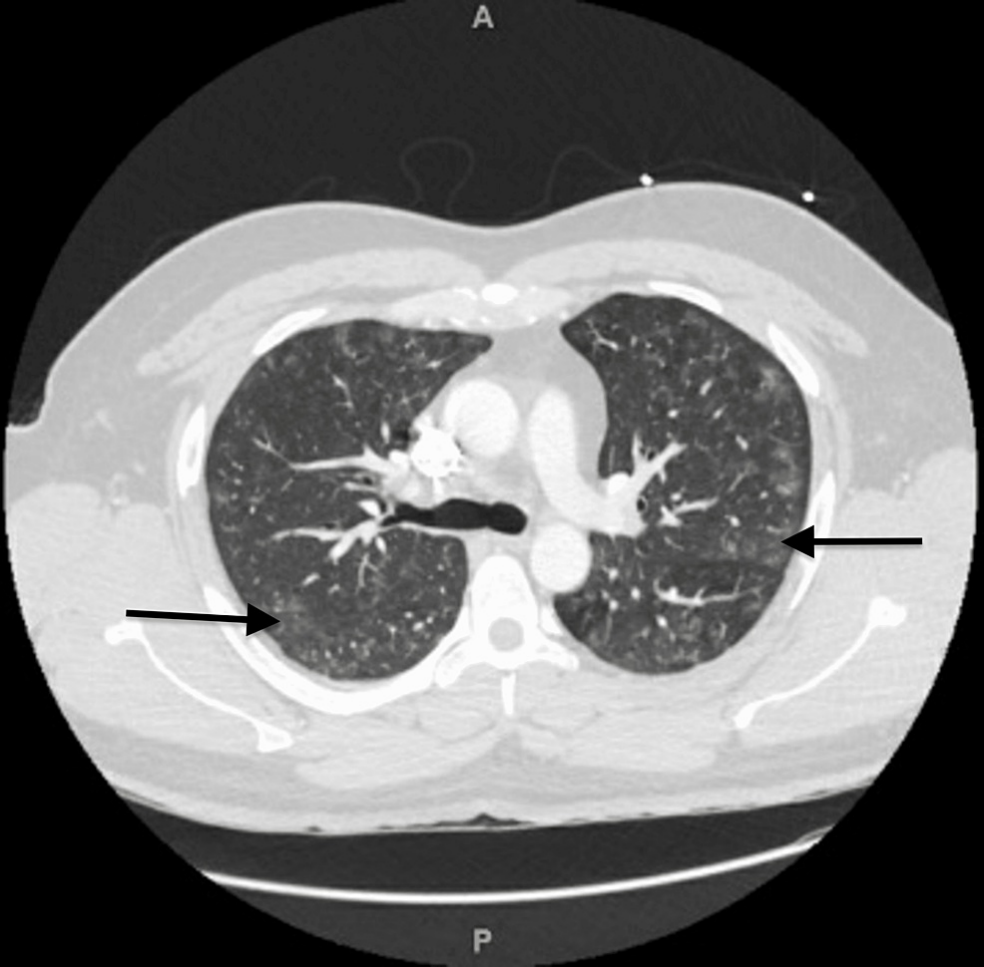
Computed tomography angiography (CTA) chest showing diffuse, bilateral ground glass lung opacities consistent with COVID-19 pneumonia.

Duplex ultrasound of bilateral extremities ruled out deep vein thromboses. A computed tomography angiography of the chest ruled out pulmonary embolism but revealed multifocal ground glass opacities from COVID-19 pneumonia (Figure 2). An echocardiogram revealed normal systolic function, left ventricular ejection fraction of 55-60%, no regional wall motion abnormalities and a negative bubble study.

He received dexamethasone 6 mg intravenously (IV) daily for COVID-19 treatment as per the hospital protocol. It is to be noted that the patient was on room air and exhibited no symptoms of COVID-19 pneumonia.

Given a major thrombotic event in the setting of COVID-19 infection in an otherwise healthy individual, a multidisciplinary discussion was held with cardiology and hematology. Decision was made to initiate him on anticoagulation with unfractionated heparin and he was bridged to warfarin. Patient continued to be on dual antiplatelet therapy (DAPT) and statins. Literature review also revealed reports of intracardiac thrombus with COVID-19 infection, and a plan was thereby made to perform transesophageal echocardiography. However, patient refused. As his clinical condition dramatically improved, he was discharged on DAPT, warfarin, high-dose statin, beta-blockers and angiotensin converting enzyme inhibitors (ACEI).

## Discussion

COVID-19 infection is an entity that usually presents with respiratory and systemic symptoms including; shortness of breath, fever, anorexia, myalgias, headache, fatigue, non-productive cough, sore throat and rhinorrhea. The most common sequelae of the infection that results in in-hospital admissions are viral pneumonia and acute respiratory distress syndrome.

These patients are also at an increased risk of thrombosis [3]. Thrombotic events occur in about 5-23% of cases. Presentations include pulmonary embolism, stroke, deep vein thrombosis and myocardial infarction (MI). A study of patients hospitalized with COVID-19 across four New York City hospitals showed that up to 16% of patients sustained a thrombotic event [4]. 8.9% of those patients presented with MI. However, the patient population that sustained MI had high risk factors or had a pre-existing coronary artery disease. Another study reported incidence of arterial thromboemboli during hospitalization was 0.13% in patients who tested positive for COVID-19 and 0.19% in patients who tested negative. However, the study concluded the presence of a composite metabolic syndrome profile to be associated with arterial clot formation [5]. Review of literature showed a rise in cases of acute coronary syndrome (ACS) in young patients with a low-risk profile in setting of COVID-19 pneumonia [6-8].

The mechanisms that underlie cardiovascular disease in COVID-19 patients are not completely understood. The severe acute respiratory syndrome coronavirus 2 (SARS-CoV-2) infects cells via the angiotensin converting enzyme 2 receptor (ACE2R), which is expressed in cells across many organs, including endothelial cells in vascular tissue [9]. Endothelial cell infection by the SARS-CoV-2 causes endotheliitis, a diffuse endothelial inflammation that instigates endothelial dysfunction that is associated with cell apoptosis. This inflammatory process causes microvascular disruptions that result in vasoconstriction, tissue edema and a procoagulant state that can result in organ ischemia [10]. This in addition to COVID-induced cytokine storm, hypoxic injury, and hypercoagulability predisposes patients to a thrombotic event. The sequence of events would be responsible for acute coronary syndromes in patients with pre-existing disease or endorsing high risk factors but can be significant enough to cause an acute event in otherwise healthy patients as in our case.

When considering the management of ST-elevation myocardial infarction (STEMI) in patients with suspected or positive COVID-19 infection, it is important to note that COVID-19-associated myocarditis also can present with ST-segment elevation. It is critical to differentiate these conditions as the management varies and these subjects should be carefully selected for invasive methods of revascularization and reperfusion [11].

Algorithms have been put forward that might assist clinicians in the triage and decision-making process when deciding to whom and how resources are to be allocated [12]. If there is a high index of suspicion for an ongoing acute coronary process, then the management follows the same lines regardless of COVID-19 infection, with rapid activation of cardiac catheterization laboratory for percutaneous coronary intervention.

It is important for all medical personnel involved to employ precautions against airborne, droplet and contact transmission of the virus including the use of personal protective equipment (PPE) and the proper sanitation of the medical equipment and environment in order to decrease the risk of transmission within the hospital and in the community at large [13].

## Conclusions

Acute coronary syndrome must be considered in the differential diagnosis in patients with COVID-19 infection presenting with acute chest pain, regardless of their age and cardiovascular risk factors. We need a high index of suspicion to identify these patients and triage them in the right direction. The mechanism that underlies the cardiovascular disease involves entry of the COVID-19 virus via ACE2 receptors into endothelial cells of vascular tissue. The resulting diffuse inflammation results in vasoconstriction, tissue edema and a procoagulant state. This in addition to cytokine storm, hypoxic injury and hypercoagulability induced by COVID-19 predisposes patients to a thrombotic event. The management of ACS in patients with COVID-19 infection follows the same pathways as those without COVID-19 infection. However, it is essential to differentiate COVID-19-associated ACS from COVID-19-induced myocarditis to reduce the risk of unwarranted invasive procedures and possible complications.
